# Supplementary material for: A Critical View of the Application of the APEX Software (Aqueous Photochemistry of Environmentally-Occurring Xenobiotics) to Predict Photoreaction Kinetics in Surface Freshwaters
Source: Molecules. 2019 Dec 18;25(1):9. doi: 10.3390/molecules25010009 (PMC7017383; doi:10.3390/molecules25010009)
Supplement: Supplementary file 1 [file molecules-25-00009-s001.zip › 3.2.4_gcc-4.4.0.zip!/3.2.4_gcc-4.4.0/3.2.4_gcc-4.4.0/doc/octave/html/liboctave/Nonlinear-Constraints.html]

Nonlinear Constraints - Octave C++ Classes


Next: Quadratic Programming,
Previous: Linear Constraints,
Up: Optimization


---

### 9.4 Nonlinear Constraints

— : **NLConst** (void)  
— : **NLConst** (int n)  
— : **NLConst** (const ColumnVector lb, const NLFunc f, const ColumnVector ub)  
— : **NLConst** (const NLConst &a)

— : NLConst& **operator =** (const NLConst &a)
